# Unexpected Cow’s Milk Proteins in a “Vegan” Easter Egg as a Cause of Anaphylaxis

**DOI:** 10.3390/foods14101737

**Published:** 2025-05-14

**Authors:** Corinne Bani, Patrizia Restani, Salvatore Tripodi, Francesca Mercogliano, Francesca Colombo, Chiara Di Lorenzo

**Affiliations:** 1 Department of Pharmacological and Biomolecular Sciences, Università degli Studi di Milano, 20133 Milan, Italy; corinne.bani@unimi.it (C.B.); francesca.mercogliano@unimi.it (F.M.); francesca.colombo1@unimi.it (F.C.); chiara.dilorenzo@unimi.it (C.D.L.); 2 Faculty of Pharmacy, Università degli Studi di Milano, 20133 Milan, Italy; 3 Coordinated Research Center (CRC) “Innovation for Well-Being and Environment” (I-WE), Università degli Studi di Milano, 20122 Milan, Italy; 4 Allergology Service, Policlinico Casilino Hospital, 00169 Rome, Italy; salvatore.tripodi@gmail.com

**Keywords:** milk proteins, food allergy, SDS-PAGE, ELISA assay

## Abstract

Background: Cow’s milk is the most frequent cause of food allergies in children, with caseins and β-lactoglobulin being considered the main allergens. Concerningly, numerous international agencies have highlighted a growing risk of allergic reactions in milk-allergic individuals after the consumption of products labelled as “vegan”. Objectives: We describe the case of a 3.5-year-old boy with a history of a food allergy to milk who complained of anaphylactic clinical symptoms after eating a vegan Easter egg. The aim of this study was to confirm the cause of the clinical symptoms, searching for the possible presence of milk proteins in the vegan chocolate. Methods: An experimental approach based on electrophoretic (SDS-PAGE) and immunoenzymatic techniques (ELISA) was applied. Results: SDS-PAGE indicated the presence of milk proteins, which was confirmed and quantified via ELISA (3034 ± 115 mg/kg). Conclusions: The data obtained demonstrate that the severe clinical symptoms were due to the unexpected presence of milk proteins in a vegan product, underlining the critical need for rigorous allergen quality control throughout the food industry.

## 1. Introduction

In recent decades, concern regarding food allergies has grown substantially among consumers, families, clinicians, and policymakers [1]. Food allergies are recognized as frequent conditions, with rates reaching up to 10% among infants in some countries [2]. In Europe, the prevalence of food allergies among school-age children is estimated to range from 1% to 5.6%, while food sensitization can affect up to 28.7% of this population [3]. Individuals with allergies to animal proteins (e.g., milk, eggs, etc.) may consider “vegan” labels to be a marker of safety since the term “vegan” typically indicates that a product does not contain ingredients of animal origin, such as meat, poultry, fish, seafood, eggs, and milk [4]. The market availability of prepackaged foods labeled as “vegan” has significantly increased in the last few decades, raising the chances of unexpected allergic reactions [5]. According to Spolidoro et al. (2023), the prevalence of food allergies in Europe, confirmed through food challenge tests, allows for the delineation of specific percentages for various allergens: cow’s milk (0.3%), eggs (0.8%), wheat (0.1%), soy (0.3%), peanuts (0.1%), tree nuts (0.04%), fish (0.02%), and shellfish (0.1%) [6].

In the case of children, cow’s milk is one of the most frequent causes of anaphylaxis [7]. Cow’s milk contains two main protein fractions, namely, caseins (alphaS1-, alphaS2-, beta-, and kappa-caseins) and whey proteins (alpha-lactalbumin (α-LA), beta-lactoglobulin (β-LB), bovine lactoferrin, bovine serum albumin (BSA), and bovine immunoglobulins), constituting 80% and 20% of the total proteins, respectively [8]. While most patients with a cow’s milk allergy show sensitization to multiple milk proteins, certain proteins, such as caseins and β-LB, are more frequently associated with clinical symptoms [8]. Allergic reactions can range in terms of the severity of symptoms from hives to facial swelling, skin redness, gastrointestinal disturbances, and fatal anaphylaxis [5]. In individuals with a milk allergy, anaphylaxis, a severe and potentially life-threatening reaction, may occur either immediately (less than 2 h after ingestion) or at later period [9]. Given the necessity of the strict dietary avoidance of allergens in clinical management, patients and their families are advised to strictly scrutinize food labels for allergen content. European regulation 1169/2011 requires the clear indication of food allergens on labels [10]. In addition, Regulation (EC) No. 178/2002 (General Food Law) requires that any hazard resulting from the unintentional presence of certain substances in food, such as through cross-contamination, must be addressed in compliance with its provisions [11,12]. The mandatory indication of allergenic ingredients on labels is a very useful tool allowing patients to avoid consuming foods responsible for clinical reactions. On the other hand, it is not uncommon for labeling to be inadequate, consequently posing a risk for allergic patients or, on the contrary, leading to pointless abstention from foods in which the possible presence of allergens is declared for precautionary purposes only [13]. The inclusion of undeclared allergens in the RASFF system reflects the acknowledgment that these allergens pose a public health risk for the corresponding population [14]. Established by the European Commission, the RASFF system database serves to track the latest information regarding food recalls and public health alerts across all European Union (EU) countries as well as Norway, Liechtenstein, Iceland, and Switzerland [15]. Notably, from 2018 to 2021, the Rapid Alert System for Food and Feed (RASFF) documented 844 notifications of the presence of undeclared food allergens, with milk being the most frequently cited allergen (20.5% of notifications), including in cocoa products. Alarmingly, 4.4% of these notifications pertained to products labeled as “vegan”, wherein milk was the involved allergen in 56.8% of cases [15]. The issue of undeclared milk allergens is also underscored by the US Food and Drug Administration (FDA). A survey conducted on dark-chocolate samples in 2018–2019 revealed that 88 out of 119 samples analyzed contained milk or its derivatives without being properly labeled, posing severe risks to allergic individuals [16,17]. Some of them contained milk levels as high as 3400 ppm (mg/kg), posing severe risks to allergic individuals. Similarly, Manny et al. (2021) showed that 148 out of 159 samples analyzed were positive for milk proteins [18], and Bedford et al. (2017) confirmed that a high percentage of dark-chocolate-based products can contain milk at concentrations high enough to induce allergic reactions in the most sensitive subjects [19]. These findings are particularly concerning considering the increasing incidence of cow’s milk allergies in adults and especially children [20]. Despite the prevalence of “contaminated” chocolate samples, allergic reactions specifically due to chocolate are rarely reported, and only in a few cases have provided documentation supporting the IgE-mediated allergy of a patient (via an oral challenge) or the chemical analysis of a sample consumed [21]. In this study, we aimed to investigate whether “vegan” chocolate (VEC) was contaminated by milk proteins, allowing us to confirm whether such proteins were the cause of the reported clinical reaction in a boy.

## 2. Materials and Methods

### 2.1. Case Report

To underline the risk of allergic reactions due to undeclared milk allergens, we report the case of a 3.5-year-old boy with a documented history of food allergy to milk dating to the first year of his life. The child, who had been exclusively breastfed, developed allergic proctocolitis 15 days after birth. Despite being kept on a special diet recommended by the pediatrician, the child manifested atopic dermatitis after one month. At 6 months of age (while the child was being weaned), the mother introduced fruits, meat, and Parmesan cheese into the child’s diet; one month later, perioral urticaria appeared. Diagnostic tests were used to search for possible food allergies. Upon conducting the skin prick test (SPT), the following positive reactions were exhibited in terms of wheal diameter: cow’s milk, 17 mm, and parmesan, 7 mm. The results of an ImmunoCAP allergen test (Thermo Fisher Scientific Inc., Monza, Italy) showed that the child was sensitized against several milk allergens; the levels of specific IgEs, expressed as kUA/L, were as follows: alpha-lactalbumin, 77.6; beta-lactoglobulin, 12.5; and caseins, 20.9. The child was diagnosed with a milk allergy and prescribed a diet free from milk and derivatives. Two years later, immediately after eating an Easter egg produced using a so-called “vegan” chocolate, the child complained of abdominal pain, a cough, urticaria, ear angioedema, hoarseness, difficulty swallowing, rhinorrhea, and bronchospasms. In order to investigate the issue and publish data, we obtained informed consent from the parents of the allergic child.

In this context, this study was conducted to verify whether potential contamination of the “vegan” chocolate (VEC) by milk proteins was responsible for inducing clinical symptoms in the child. 

### 2.2. Samples

This study included different samples for comprehensive analysis, including (1) the residual portion of the vegan Easter egg chocolate consumed by the child. The label on the Easter egg package reported the product was “chocolate without lactose”, indicating its suitability for individuals who are lactose-intolerant or prefer lactose-free options. The ingredient list included sugar, cocoa butter (25%), cocoa powder (14%), rice flour (rice syrup), sunflower lecithin as an emulsifier, and vanilla flavoring. The label indicated that the product may contain traces of nuts and gluten, but it made no reference to the possible presence of milk. The child consumed only a portion of the Easter egg, and the remaining part was left to our disposal for chemical analysis. The other samples were (2) commercially available milk chocolate and (3) chocolate with 90% cocoa from the market. Along with the chocolate samples, analyses were performed on the following matrices: (1) rice flour, as an ingredient of the vegan Easter egg; (2) isolated rice proteins; and (3) freeze-dried milk. These additional samples were used to better identify protein bands present in the electrophoretic analysis.

### 2.3. SDS-PAGE

Chocolate samples were prepared by following the method described by Hemmati and Keeney (1979) [22], with slight modifications [23]. To optimize protein loading for SDS-PAGE, 40 mg of commercial chocolates and 80 mg of “vegan” Easter egg chocolate (VEC) were melted at 65 °C. Following the melting process, the chocolate aliquots were mixed with sample buffer (0.125 M Tris-HCl at pH 6.8, 3.75% glycerol, 1% SDS, and 2.5% β-mercaptoethanol), stirred for 2 h, and centrifuged at 15,000× *g* for 2 min at room temperature using an Avanti J-25 centrifuge (Beckman Coulter, Brea, CA, USA). The resulting supernatant was separated and stored at −20 °C until further analysis. Additionally, the freeze-dried milk sample and rice flour were suspended in sample buffer to attain concentrations of 10 mg/mL and 20 mg/mL, respectively. Isolated rice proteins and standards of α-casein, β-casein, α-lactalbumin, and β-lactoglobulin (Sigma-Aldrich, Darmstadt, Germany) were prepared in the sample buffer at a concentration of 1 mg/mL. A pre-stained molecular weight standard solution (Bio-Rad, Richmond, CA, USA) was included and analyzed alongside the samples. SDS-PAGE was conducted as described by Ballabio and coworkers [23]. In the SDS-PAGE method, SDS anions bind to the proteins present in samples, creating SDS–protein complexes characterized by a uniform and high negative charge density, thereby separating proteins according to their molecular weight [24]. A gradient polyacrylamide gel (9–19%) was employed. The electrophoresis was carried out at a voltage of 90 V for approximately 6 h using a Power Supply (model E 835, 300 V, 500 mA, Consort Belgium, Brussels, Belgium) at room temperature [25]. Following electrophoresis, the gel was immersed in a 20% trichloroacetic acid (TCA) solution (Sigma-Aldrich, Steinheim, Germany) for 20 min to precipitate the proteins. Protein bands were detected using Coomassie Brilliant Blue G-250 (Bio-Rad, Hercules, CA, USA), and the background was destained using a solution composed of 10% acetic acid and 20% ethanol (both from VWR International, Fontenay-sous-Bois, France), supplemented with activated charcoal (Merck, Darmstadt, Germany).

### 2.4. ELISA Analysis

The ELISA (Enzyme Linked Immunosorbent Assay) technique was used to verify the presence of milk proteins in the samples and perform relative quantification. For this purpose, the RIDASCREEN FAST Milk kit (Nr. R4652, R-Biopharm Italia Srl, Melegnano, Italy) was utilized; it was specifically developed for the quantification of milk proteins in food matrices [26]. The proteins detected were caseins and β-lactoglobulin, which are the most important allergens in milk [8,27]. The samples were prepared, treated, and analyzed following the official method described by the producer. Quantification was performed using a spectrophotometer (VICTOR X3; PerkinElmer, Milano, Italy) at 450 nm. To ensure accuracy and reliability, all samples were analyzed in three independent repetitions, thereby enhancing the robustness of the data obtained from the ELISA analysis.

All data were reported as the means ± standard deviations (SDs).

## 3. Results and Discussion

### 3.1. SDS-PAGE

**Figure 1:**
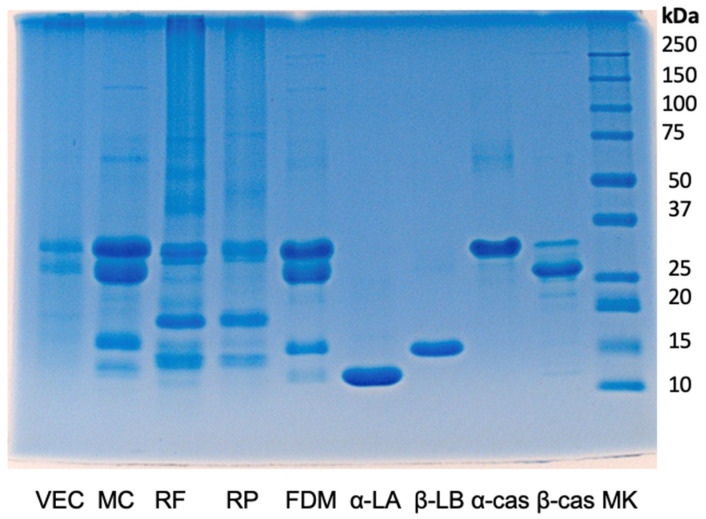
SDS-PAGE results for the samples included in this study. VEC: “vegan” Easter egg chocolate; MC: commercial milk chocolate; RF: rice flour; RP: rice proteins; FDM: freeze-dried milk; α-LA: α-lactalbumin; β-LB: β-lactoglobulin; α-cas: α-casein; β-cas: β-casein; MK: markers of molecular weights.

Figure 1 illustrates the results of the SDS-PAGE (Sodium Dodecyl Sulfate Polyacrylamide Gel Electrophoresis) performed on the samples analyzed in this study.

The commercial milk chocolate (MC) and freeze-dried milk (FDM) show bands corresponding to the four main cow’s milk proteins: α-casein, β-casein, β-lactoglobulin, and α-lactalbumin [28]. Within the 15–35 kDa range, the VEC shows two main bands with the same electrophoretic run of α- and β-caseins and a minor one corresponding to β-lactoglobulin.

The prevalence of casein bands may be attributed to their high relative abundance in cow’s milk, where caseins constitute up to 80% of the total protein content, while whey proteins account for the remaining 20% [28]. The prevalent presence of casein bands in the VEC sample could also suggest cross-contamination from food processes where caseinates are often used instead of whole milk. Regarding whey proteins, β-lactoglobulin is the predominant component, making up approximately 50% of this fraction [28].

Although rice is an ingredient in vegan chocolate, only bands with very light intensity were attributable to it in the VEC run (see the corresponding bands in RF and RP).

These findings indicate the presence of milk proteins in the vegan chocolate consumed by the patient, for which confirmation via an ELISA test was necessary. Overall, the findings underscore the complexity of food composition analysis and the importance of employing comprehensive approaches to ensure product integrity and safety.

### 3.2. ELISA Analysis

To determine whether milk proteins were present in the VEC and, if so, quantify them, the ELISA (Enzyme-Linked Immunosorbent Assay) technique was applied [26]. According to the information provided by the producer, RIDASCREEN FAST Milk kit (Nr. R4652, R-Biopharm Italia Srl, Melegnano, Italy) was developed to detect the main milk allergens in food (caseins and β-lactoglobulin). The degree of spectrophotometric absorption measured at the end of analytical steps is proportional to the milk protein concentration in the samples, which is calculated in mg/kg by using a semilogarithmic calibration curve; the corresponding data are provided in Table 1.

As expected, milk proteins were undetectable (<LOD 0.7 mg/kg) in the dark chocolate and present in the commercial milk chocolate at a concentration of 47,156 ± 6460 mg/kg. This value agrees with the milk percentage required by Directive 2000/36/EC for this product (i.e., no less than 14% dry milk solids obtained by partly or wholly dehydrating whole milk, semi- or full-skimmed milk, or cream or from partly or wholly dehydrated cream, butter, or milk fat) [29]. Significant amounts of milk proteins were quantified in the vegan Easter chocolate (VEC): 3034 ± 115 mg/kg. The unexpected presence of milk proteins in a product marketed as vegan is of particular concern because this product, although intended for another category of consumers, can be considered safe by subjects allergic to milk, posing very serious potential clinical consequences. This finding raises questions about the adequacy of risk assessment and mitigation strategies for allergic individuals normally applied in food industries.

## 4. Conclusions

The combined use of SDS-PAGE and ELISA provided robust evidence of the presence of milk protein in VEC, indicating its involvement in the clinical reactions observed in the child considered in this study. The unexpected and significant presence of milk proteins in vegan Easter egg chocolate (VEC) indicates there is a significant risk for individuals with milk allergies and emphasizes the necessity of thorough ingredient screening and accurate labeling. This discrepancy between the expected and analytically detected milk protein content serves as a cautionary tale, urging the need for stricter quality control, improved manufacturing practices, and heightened consumer awareness. The results from this study are in agreement with some previous studies wherein cases of clinical symptoms caused by vegan products were reported [30,31].

Initiatives from organizations like the Food Standards Agency in the UK [16,30] highlight the importance of effective communication in informing producers and educating the public about the specific meaning of labelling. By addressing these challenges, we can better protect vulnerable populations from potential allergic reactions.

## Figures and Tables

**Table 1:** Milk protein content in VEC and commercial chocolate.

Sample	Milk Protein Concentration (mg/kg)	
	Values	Mean ± SD
Vegan Easter egg chocolate (VEC)	2935	3034 ± 115
	3005	
	3160	
Commercial 90% dark chocolate	N.D.	N.D.
	N.D.	
	N.D.	
Commercial milk chocolate	45,348	47,156 ± 6460
	54,326	
	41,791	

N.D. = <LOD 0.7 mg/kg.

## Data Availability

The original contributions presented in the study are included in the article, further inquiries can be directed to the corresponding author.

## Abbreviations

The following abbreviations are used in this manuscript:

α-LA	α-lactalbumin
β-LB	β-lactoglobulin
α-cas	α-casein
β-cas	β-casein
BSA	bovine serum albumin
LD	linear dichroism
SPT	skin prick test
VEC	“vegan” easter egg chocolate
TCA	trichloroacetic acid
MC	commercial milk chocolate
RF	rice flour
RP	rice proteins
FDM	freeze-dried milk
MK	markers of molecular weights
